# Polyamines function in stress tolerance: from synthesis to regulation

**DOI:** 10.3389/fpls.2015.00827

**Published:** 2015-10-13

**Authors:** Ji-Hong Liu, Wei Wang, Hao Wu, Xiaoqing Gong, Takaya Moriguchi

**Affiliations:** ^1^Key Laboratory of Horticultural Plant Biology, College of Horticulture and Forestry Science, Huazhong Agricultural University, Wuhan, China; ^2^State Key Laboratory of Crop Stress Biology for Arid Areas, College of Horticulture, Northwest A&F University, Yangling, China,; ^3^National Institute of Fruit Tree Science, National Agriculture and Food Research Organization, Tsukuba, Japan

**Keywords:** abiotic stress, antioxidant, polyamine, polyamine biosynthesis, ROS, transcriptional regulation

## Abstract

Plants are challenged by a variety of biotic or abiotic stresses, which can affect their growth and development, productivity, and geographic distribution. In order to survive adverse environmental conditions, plants have evolved various adaptive strategies, among which is the accumulation of metabolites that play protective roles. A well-established example of the metabolites that are involved in stress responses, or stress tolerance, is the low-molecular-weight aliphatic polyamines, including putrescine, spermidine, and spermine. The critical role of polyamines in stress tolerance is suggested by several lines of evidence: firstly, the transcript levels of polyamine biosynthetic genes, as well as the activities of the corresponding enzymes, are induced by stresses; secondly, elevation of endogenous polyamine levels by exogenous supply of polyamines, or overexpression of polyamine biosynthetic genes, results in enhanced stress tolerance; and thirdly, a reduction of endogenous polyamines is accompanied by compromised stress tolerance. A number of studies have demonstrated that polyamines function in stress tolerance largely by modulating the homeostasis of reactive oxygen species (ROS) due to their direct, or indirect, roles in regulating antioxidant systems or suppressing ROS production. The transcriptional regulation of polyamine synthesis by transcription factors is also reviewed here. Meanwhile, future perspectives on polyamine research are also suggested.

## Introduction

As sessile organisms, plants are frequently challenged by a variety of adverse biotic or abiotic environmental factors. Since, unlike animals, plants cannot escape from unfavorable environments, harsh stresses constitute major factors that limit growth and development, and severely restrict the production of high-quality agricultural crops. Exposure to the stressful conditions can therefore lead to a substantial difference in potential and actual crop yields, the size of which largely depends on the severity and duration of the environmental stresses in question. Abiotic stresses, such as drought, flooding, extreme temperatures, high salinity, chemical toxicity, nutrient deficiency and others, are regarded as the predominant causes of crop loss and may account for more than 50% reduction of the yield of the major annual and perennial crops worldwide (Wang et al., 2003). In this regard, understanding how plants adapt to, and survive, the abiotic stresses is important for the efficient exploitation of genetic resources with desirable stress tolerance, and for developing new approaches to enhance stress tolerance.

Plant evolution has been accompanied by the development of complex and highly coordinated systems that allow adaptation to the stresses, involving signaling cascades that start with signal perception, and result in a variety of stress responses. Recently, significant progresses have been made in elucidating the molecular and genetic pathways involved in stress responses, and a number of key components in the stress signaling cascade events have been identified (Yuan et al., 2013; Wisniewski et al., 2014; Gehan et al., 2015; Shi et al., 2015). In the signal transduction pathway, stress signals are perceived by sensors that are primarily located at the plasma membrane, resulting in the release or activation of various secondary messengers, such as calcium (Ca), ROS (reactive oxygen species), and inositol phosphates, which relay the stress signals and activate downstream components, such as protein kinases and protein phosphatases (Nakashima et al., 2009; Danquah et al., 2014; Liu et al., 2014a; Ma et al., 2015). These proteins orchestrate the balance of protein phosphorylation and play a key role in the regulation of transcription factors (TFs), which bind to *cis*-acting elements in the promotes of their downstream target genes, thereby activating their transcription. This signaling cascade has been shown to be conserved in various plants and allows the plants to survive under the harsh environments (Liu et al., 2014a).

Stress responses are manifested by a range of morphological, physiological, biochemical, and molecular changes. Among these, molecular reprogramming plays a pivotal role, and a large number of studies have described the up- or down-regulation of a wide spectrum of stress-responsive genes (Seki et al., 2002; Thomashow, 2010; Liu et al., 2014a). These genes are generally classified into regulatory or functional types, based on the function of their products. Regulatory genes, encoding protein kinases, phospholipases, and TFs, act as master switches involved in hierarchical signaling cascades, thereby playing vital roles in transcriptional control of downstream stress-responsive genes. The functional genes act directly to mitigate stress-derived injuries via their products, which include a diverse set of metabolites (Shinozaki and Yamaguchi-Shinozaki, 2007). These protective approaches include the stabilization of membranes and macromolecules, alleviation of oxidative stresses, and maintenance of water status. One well studied group of metabolites comprises the polyamines, which have long been suggested to protect and maintain the function and structure of cellular components in response to stresses. Since the first report describing the accumulation of the polyamine putrescine as a result of potassium deficiency (Richards and Coleman, 1952), a large number of studies have implicated polyamines in plant responses to a myriad of abiotic stresses, and these have been reviewed elsewhere (Liu et al., 2007; Kusano et al., 2008; Alcázar et al., 2010a; Hussain et al., 2011; Minocha et al., 2014; Shi and Chan, 2014; Tiburcio et al., 2014). Here, we review recent progress in understanding the association between polyamines and stress responses, with an emphasis on their role in maintenance of ROS homeostasis. In addition, recent advances in identifying and characterizing upstream regulatory genes involved in the stress-induced transcriptional regulation of polyamine metabolism are also discussed.

## Polyamine Synthesis and Catabolism: Current Status

**FIGURE 1:**
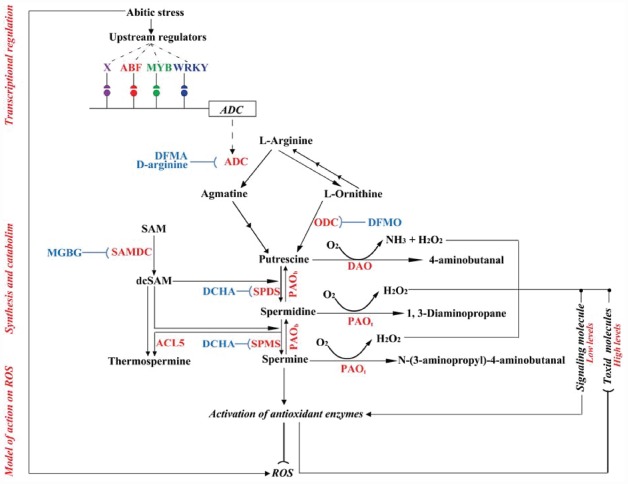
**A schematic diagram on synthesis, catabolism, regulation, and action of plant polyamines under abiotic stresses.** The enzymes are shown in red, while the inhibitors of the polyamine biosynthetic enzymes are shown in blue. PAO_*t*_ indicates the PAO simplicated in terminal metabolism, whereas PAO_*b*_ is involved in back conversion. Circles indicate the relevant *cis*-acting elements within the ADC promoter. *ADC* coding sequence is boxed, and the promoter is shown by the horizontal line. X is an unknown transcription factor that regulates the expression of *ADC* gene. Arrows mean promotion or stimulation, whereas blunted arrows indicate inhibition, of the related processes. The dashed arrow shows the translation from the *ADC* gene to ADC protein/enzyme.

Polyamines (PAs) are low-molecular-weight, aliphatic polycations that are ubiquitously distributed in all living organisms, including bacteria, animals, and plants (Hussain et al., 2011). There are three major PAs in plants, putrescine (Put), spermidine (Spd), and spermine (Spm), although other types of PAs, such as cadaverine, can also be present. The plant PA biosynthetic pathway has been extensively studied (Kusano et al., 2008; Vera-Sirera et al., 2010; Pegg and Casero, 2011; Gupta et al., 2013) and differs from that of animals in that it involves two precursors, l-ornithine and l-arginine, to generate putrescine, while only l-ornithine is used in animals. In plants, Put is produced via the catalytic actions of ornithine decarboxylase (ODC, EC 4.1.1.17) and arginine decarboxylase (ADC, EC 4.1.1.19) in three steps. Put is then converted into Spd by Spd synthase (SPDS, EC 2.5.1.16), with the addition of an aminopropyl moiety donated by decarboxylated *S*-adenosylmethionine (dcSAM). dcSAM is synthesized from methionine via two sequential reactions that are catalyzed by methionine adenosyltransferase (EC 2.5.1.6) and *S*-adenosylmethionine decarboxylase (SAMDC, EC 4.1.1.50), respectively. Spd is then converted into Spm or thermospermine, again using dcSAM as an aminopropyl donor, in a reaction catalyzed by Spm synthase (SPMS, EC 2.5.1.22) and thermospermine synthase (ACL5, EC 2.5.1.79), respectively (Figure 1). It should be noted that there is no known gene encoding ODC in the sequenced genome of model plant *Arabidopsis thaliana* (Hanfrey et al., 2001), suggesting that this species may only produce Put via the ADC pathway. Finally, PA synthesis may vary between tissues/organs, one example being that the shoot apical meristem of tobacco (*Nicotiana tabacum*) serves as the predominant site of Spd and Spm synthesis, while Put is mostly synthesized in roots (Moschou et al., 2008).

Apart from their *de novo* synthesis, PAs have been shown to undergo catabolism (Figure 1), catalyzed by two classes of enzymes, copper-containing diamine oxidases (CuAOs) and FAD-containing polyamine oxidases (PAOs; Cona et al., 2006). CuAOs mainly catalyze the oxidation of Put and cadaverine (Cad) at the primary amino groups, producing 4-aminobutanal, peroxide (H_2_O_2_) and ammonia (Alcázar et al., 2010a; Moschou et al., 2012). Generally, CuAO proteins exhibit high affinity for Put and Cad than for Spd and Spm (Moschou et al., 2012), although it has been demonstrated that *A. thaliana* CuAO enzymes can also use Spd as a substrate (Planas-Portell et al., 2013). Plant CuAO proteins are present at high level in dicots, especially in Fabaceous species, such as pea, chickpea, lentil, and soybean seedlings (Cona et al., 2006). Until now, *CuAO* genes have been identified in only a few plant species, such as *A. thaliana* (Møller and McPherson, 1998; Planas-Portell et al., 2013) and chickpea (Rea et al., 1998). In *A. thaliana* there are at least 10 putative *CuAO* genes, and four of these (AtAO1, AtCuAO1, AtCuAO2, and AtCuAO3) have also been identified (Møller and McPherson, 1998; Planas-Portell et al., 2013).

In contrast to CuAOs, PAOs are present at high levels in monocots and have a high affinity for Spd, Spm, and their derivatives (Alcázar et al., 2010a). Plant PAOs are divided into two major groups, depending on their potential functions in polyamine catabolism. The first group catalyzes the terminal catabolism of Spd and Spm to produce 1,3-diaminopropane (DAP), H_2_O_2_, and *N*-(3-aminopropyl)-4-aminobutanal (Spm catabolism), or 4-aminobutanal (Spd catabolism; Cona et al., 2006; Angelini et al., 2010; Moschou et al., 2012). The second group is responsible for PA conversions, in which Spm is converted back to Spd, and Spd to Put (Moschou et al., 2012; Mo et al., 2015). To date, *PAO* genes have been identified in several plant species, including *A. thaliana* (Fincato et al., 2011), tobacco (Yoda et al., 2006), rice (*Oryza sativa*; Ono et al., 2012), barley (*Hordeum vulgare*; Cervelli et al., 2001), maize (*Zea mays*; Cervelli et al., 2000), poplar (Tuskan et al., 2006), apple (*Malus domestica*; Kitashiba et al., 2006), cotton (*Gossypium barbadense*; Mo et al., 2015), and sweet orange (*Citrus sinensis*; Wang and Liu, 2015). Plant PAO proteins are encoded by small gene families, as revealed by analyses of fully sequences genomes. There are five, seven, and six *PAO* genes in *A. thaliana*, rice, and sweet orange, respectively. However, only few of the *PAO* genes belonging to the first group have been characterized, and to date only *ZmPAO* and *OsPAO7*, from maize and rice, respectively, have been reported to be involved in PA terminal catabolism (Cona et al., 2006; Liu et al., 2014b). In contrast, many *PAO* genes belonging to the second group have been identified. For example, all five *PAO* genes from *A. thaliana* (*AtPAO1*–*AtPAO5*) and four *PAO* genes from rice (*OsPAO1*, *OsPAO3*, *OsPAO4*, and *OsPAO5*) have been shown to be involved in the back conversion of polyamines (Tavladoraki et al., 2006; Kamada-Nobusada et al., 2008; Moschou et al., 2008; Fincato et al., 2011; Ono et al., 2012; Ahou et al., 2014; Liu et al., 2014b), and recently, a sweet orange *PAO* gene (*CsPAO3*) was added to the list of identified genes from this group (Wang and Liu, 2015). As PA catabolism gives rise to the production of H_2_O_2_, which may act either as a signaling molecule at low levels or as a toxic compound when the level is high, the ratio of PA catabolism to biosynthesis has been considered as a crucial factor for induction of tolerance responses or plant cell death under abiotic stress (Moschou et al., 2008). This suggests that PA-derived H_2_O_2_ may play a key role in the maintenance of ROS homeostasis, depending on its cellular levels.

## Involvement of Polyamine Metabolism in Stress Response

### Change in PA Levels and Transcript Levels of Genes Involved in Polyamine Metabolism Under Abiotic Stress Conditions

Polyamines have been shown to be involved in various processes associated with plant growth and development, such as embryogenic competence (Silveira et al., 2013), programmed cell death (Kim et al., 2013), fruit ripening (Gil-Amado and Gomez-Jimenez, 2012), xylem differentiation (Tisi et al., 2011), as well as biofilm formation (Lee et al., 2009). Accumulating evidences suggest that plant PAs function in adaptive responses to various environmental stresses, and this is supported by the extensive variation in polyamine levels under stress conditions. Since the first report described Put accumulation under potassium deficiency decades ago (Richards and Coleman, 1952), changes in PA levels have been universally observed in various plant species subjected to a range of abiotic stresses, including drought, high salinity, low and high temperatures, nutrient deficiency, and others (Liu et al., 2007). In some cases, it has been observed that the three most abundant PAs, Put, Spd and Spm, show substantial increases in abundance following abiotic stress (Yang et al., 2007). However, in most cases, only one type of the three PAs shows a significant increase. For example, when apple callus was treated with salt, Put levels increased, while those of Spd and Spm underwent only minor changes (Liu et al., 2006). In contrast, sweet orange callus was reported to show predominant increases in Spd content when exposed to salt and cold stress conditions (Wang and Liu, 2009), and grape (*Vitis vinifera*) plants showed a dramatic accumulation of Spd and Spm following salt stress (Ikbal et al., 2014). In another study, it was reported that 18 rice varieties exhibited notable changes in Spm levels when grown under long-term drought stress (Do et al., 2014). These findings suggest that PA accumulation is influenced by different factors, such as plant species in question, stress tolerance capacity, stress types and conditions, and the physiological status of the examined tissues/organs. It also indicates the existence of complicated PA dynamics under abiotic stress, which may explain why differing or contradictory results have been reported. The size of PA pool can be correlated with the stress tolerance capacity, further underlining the significance of PAs in providing protection against stresses. Generally, tolerant genotypes accumulate greater amounts of PAs than sensitive genotypes (Hatmi et al., 2015); however, genotypes with contrasting stress tolerance have been shown to display different patterns of PA accumulation under some abiotic stresses. In several studies tolerant genotypes accumulated more Spd and Spm, while the sensitive genotypes from the same plants species accumulated more Put under the same types of stresses (Krishnamurthy and Bhagwat, 1989; Santa-Cruz et al., 1998; Liu et al., 2004). Although whether Spd and Spm play more important roles in counteracting abiotic stress remains to be determined, it is a reasonable hypothesis, since Spd and Spm contain one and two additional primary amino groups (–NH_2_), respectively, compared to Put, allowing them to be more efficient for executing protective functions.

The accumulation of PAs under abiotic stress conditions is largely due to the increased *de novo* synthesis of free PAs. Since their synthesis is primarily regulated at the transcriptional level, an understanding of the expression patterns of the biosynthetic genes is important for understanding the regulation of PA levels. To this end, a myriad of studies have been carried out to investigate the steady-state transcript levels of PA biosynthetic genes. Available data to date indicate that most of the PA biosynthetic genes, including *ADC*, *SPDS*, *SPMS*, and *SAMDC*, are up-regulated by stresses, despite a difference in the timing and degree of induction (Liu et al., 2006, 2008, 2009, 2011; Wang et al., 2011b). Of these genes, *ADC* is most widely characterized in different plants and has been demonstrated to be a crucial stress-responsive gene (Urano et al., 2004; Liu et al., 2006; Wang et al., 2011b). Increased transcript levels of the PA biosynthetic genes coincide with the accumulation of free PA in some studies, but inconsistent in others (Liu et al., 2006; Wang and Liu, 2009). One reason for the disparity between gene expression profiles and PA accumulation is likely due to PA catabolism. Notably, the expression patterns of PA biosynthetic genes have also been shown to be correlated with stress tolerance (Pillai and Akiyama, 2004). For instance, citrus genotypes with better salt and cold tolerance displayed earlier and/or greater induction of *SAMDC* transcript at the initial stages of stress treatment (Wang et al., 2010).

### Effects of Modulating PA Content on Stress Tolerance

Polyamine accumulation is usually considered to be a general plant response to abiotic stresses, but the cause-effect relationship between PA accumulation and protection remains unclear. An effective strategy for understanding the roles of PAs in stress tolerance is to modulate their cellular levels, which has been accomplished using three approaches, including their exogenous application, overexpression of their biosynthetic genes and the use of PA synthesis inhibitors.

Exogenous application of Put, Spd, or Spm at different concentrations has been shown to confer enhanced tolerance to various stresses in different plants (Duan et al., 2008). For example, exogenous application of Put considerably enhanced salt tolerance in apple callus and thermotolerance of wheat (Liu et al., 2006; Kumar et al., 2014). A recent study by Zhang et al. (2015b) demonstrated that damage caused by saline-alkaline stress to tomato (*Solanum lycopersicum*) plants was substantially alleviated when 0.25 mM Spd was applied, and exogenous Spd supplementation can also alleviate salt stress in sorghum (*Sorghum bicolor*) seedlings (Yin et al., 2015). In another study, Harindra Champa et al. (2015) demonstrated that exogenous application of 1 mM Spm reduced chilling injury during low temperature storage of grape berries, leading to maintenance of fruit quality and shelf life.

Apart from exogenous PA application, several elegant studies have shown that the overexpression of PA biosynthetic genes is an effective strategy to elevate the endogenous PA pool and to modify stress tolerance. For example, overexpression of *ADC* genes from oat (*Avena sativa*) and *Datura stramonium* resulted in greater accumulation of Put in the transgenic plants, which displayed enhanced drought tolerance when compared with the wild type (WT) genotypes (Roy and Wu, 2001; Capell et al., 2004). Recently, it was shown that constitutive overexpression of *ADC2* in *A. thaliana*, oat *ADC* in *Lotus tenuis* and *PtADC* of *Poncirus trifoliata* in tobacco and tomato noticeably increased drought tolerance in the transgenic plants (Alcázar et al., 2010b; Wang et al., 2011a; Espasandin et al., 2014). In addition, other PA biosynthetic genes, such as *ODC*, *SAMDC*, and *SPDS*, have also been overexpressed in transgenic plants, resulting in enhanced tolerance to specific stresses, such as drought and salt (Roy and Wu, 2002; Waie and Rajam, 2003). In summary, overexpression of a PA biosynthetic gene has been demonstrated to confer tolerance to various abiotic stresses (Kasukabe et al., 2004, 2006; Wi et al., 2006; Wang et al., 2011a,b), indicating that changes in the endogenous PA pool has a profound influences on stress tolerance.

Several inhibitors have been identified that repress different PA biosynthetic enzymes, thereby inhibiting endogenous PA synthesis. Their use has provided useful insights into the role of PAs in stress tolerance. Specific or non-specific inhibitors have been used in order to elucidate the role of different PAs. d-arginine, an inhibitor of ADC, was shown to be effective in reducing Put synthesis and its application to apple callus compromised salt tolerance; an effect that was reversed when exogenous Put was applied, suggesting a role for Put in combating salt stress (Liu et al., 2006). Moreover, treatment of grape plants with methylglyoxal-bis(guanylhydrazone) (MGBG), an inhibitor of SAMDC, which is involved in synthesis of Spd and Spm, led to a greater deterioration of plant growth under salt stress than those without MGBG treatment (Ikbal et al., 2014). Recently, it was shown that treatment of sorghum plants with dicyclohexylammonium sulphate (DCHA), an inhibitor of SPDS and SPMS, ameliorated the silicon-induced salt tolerance, implying the positive role of PA in this process (Yin et al., 2015).

## Role of Polyamines in Stress Tolerance: From an Antioxidant Perspective

As mentioned above, elevation of endogenous PA levels is one of the metabolic hallmarks of plants exposed to abiotic stresses (Kusano et al., 2008), implying that they are important for protecting plants against harsh environmental conditions. Nevertheless, in spite of many observations of changes in PA levels under stresses, the precise physiological and molecular mechanisms by which they confer protection remain elusive (Marco et al., 2011). The biological function of the polycationic PAs were initially associated with their capacity to bind anionic macromolecules, such as nucleic acids and proteins, a characteristic that allows PAs to play a role in the regulation of transcription and translation (Bachrach, 2010; Gill and Tuteja, 2010; Igarashi and Kashiwagi, 2010; Tiburcio et al., 2014). They have also been suggested to function in maintaining membrane stability under adverse conditions (Liu et al., 2007; Tiburcio et al., 2014); however, besides other than these mechanisms, there is increasing evidence that their role in stress tolerance is associated with modulating antioxidant systems.

Reactive oxygen species are produced under normal growth conditions, but their homeostasis is a highly coordinated balance between generation and detoxification. Under abiotic stresses, ROS production is elevated, causing excessive ROS accumulation and oxidative stress, which is toxic to living cells due to lipid peroxidation and membrane damage, and can finally result in cell death (Biswas and Mano, 2015). PAs are thought to play a role in modulating ROS homeostasis in two ways. Firstly, they may inhibit the auto-oxidation of metals, which in turn impairs the supply of electrons for the generation of ROS (Shi et al., 2010). They may also directly act as antioxidants and scavenge ROS, although there is no evidence for this mechanism at present. Secondly, PAs may affect antioxidant systems, and a number of studies have demonstrated that priming of plants with polyamines led to increases in endogenous PA contents and concomitant enhanced tolerance to abiotic stresses, such as drought, heat, and cold. The elevation of stress tolerance is concurrent with the activation of antioxidant enzymes. For example, exogenous application of Spm to *P. trifoliata* led to an elevation of POD, SOD, and CAT activities, accompanied by a remarkable decrease in ROS levels under dehydration (Shi et al., 2010). Exogenous supply of Spd to rice seedlings mitigated heat-induced damages, and increased activities of antioxidant enzymes and levels of antioxidant, accompanied by reduced accumulation of H_2_O_2_ (Mostofa et al., 2014). Similar findings have been observed using other plants, such as tobacco, soybean, cucumber, and pistachio (Xu et al., 2011; Radhakrishnan and Lee, 2013; Shu et al., 2013; Kamiab et al., 2014). On the other hand, genetic manipulation of PA biosynthetic genes has been demonstrated to promote stress tolerance via modulation of antioxidant machineries. Overexpression of *MdSPDS1* in European pear (*Pyrus communis*) resulted in an enhanced tolerance to heavy metals, which was largely ascribed to the activation of antioxidant enzymes (Wen et al., 2009). Ectopic expression of *PtADC* in tobacco and tomato also conferred enhanced dehydration and drought tolerance, coincident with a substantial repression of ROS generation in the transgenic plants (Wang et al., 2011a). Another line of evidence supporting the role of PAs in modulating ROS homeostasis is the use of inhibitors of PA biosynthetic enzymes. As an example, it was shown that the use of D-arginine resulted in a decrease in endogenous PA levels and a consequent increase in ROS accumulation (Wang et al., 2011b; Zhang et al., 2015a). These studies demonstrate that PAs may alleviate the oxidative stress of the stressed plants through regulation of antioxidant systems, along with changes in the ROS production and redox status (Shu et al., 2013; Tanou et al., 2014).

However, a direct link between increased PA levels and antioxidant enzyme activity has yet to be proven. One possibility is that the PAs may function as signaling molecules that can activate the antioxidant enzymes, and indeed Spm has been suggested to act as a signaling molecule (Mitsuya et al., 2009). Another link may be the production of H_2_O_2_ by PAO-mediated PA catabolism. An increase in the endogenous PA levels to a certain threshold may promote their degradation, generating H_2_O_2_. It is known that H_2_O_2_ plays dual roles in plant responses to abiotic stresses, one being to act as a regulator of signaling cascades at a low cytosolic concentration, which may contribute to the induction of antioxidant enzymes (Moschou et al., 2008; Zhang et al., 2015a; Figure 1). On the other hand, the PAs may influence various antioxidant enzymes through regulation of their expression. Higher transcript levels of antioxidant enzyme-encoding genes have been detected in tissues treated with exogenous PAs or in the transgenic plants overexpressing PA biosynthetic genes (Tanou et al., 2014; Zhang et al., 2015b).

## Transcriptional Regulation of Polyamine Synthesis Under Abiotic Stress

Earlier studies demonstrated that the PA biosynthetic genes display disparate expression profiles under abiotic stresses. For example, *PpADC* of peach was up-regulated by dehydration, salt, cold, and cadmium (Liu et al., 2009), but repressed by high temperature. It has to be pointed out that the PA biosynthetic genes can be differentially influenced by a particular stress, as exemplified by *MdADC1*, which is more responsive to salt stress than other PA biosynthetic genes (Liu et al., 2006). In addition, the transcript levels of PA biosynthetic genes can vary significantly between stress tolerant and stress sensitive genotypes. Such findings suggest that the PA biosynthetic genes may be under tight transcriptional regulation during abiotic stress responses, and so identification and characterization of the associated upstream transcriptional regulators will likely be important in connecting stress responses with PA metabolism.

It has been suggested that *ADC* acts as an important polyamine biosynthetic gene in response to abiotic stresses, and it has been more extensively characterized than other genes in the pathway. The expression of *ADC* genes from a number of plant species has been described; in particular those from several species, such as *A. thaliana*, *P. trifoliata*, have been functionally characterized (Urano et al., 2004; Wang et al., 2011a,b). Thus, *ADC* genes are promising candidates used for identifying potential transcriptional regulators, such as TFs or protein kinases. The identification and bioinformatics analysis of promoter sequences are common first steps toward identifying potential TFs that regulate a PA biosynthetic gene, prior to the use of yeast one-hybrid screening of cDNA libraries. This generally involves characterizing putative *cis*-acting elements that are present within the promoters. Recently, Basu et al. (2014) reported that *in silico* analysis of the promoter region of rice *SamDC* gene revealed the presence of several putative *cis*-acting elements, such as ABRE, LTRE, MYBR, and W-box, which have been shown to be closely associated with various environmental factors, such as drought, cold, and abscisic acid (ABA) signaling. These findings suggest that the PA biosynthetic genes may be controlled by a common set of TFs, or that a given TF may control different genes involved in PA biosynthesis. This idea is congruent with earlier reports that PA biosynthetic genes, such as *PpADC* (Liu et al., 2009) or *PtADC* (Wang et al., 2011b), are responsive to different stresses. In addition, it also suggests that the endogenous PA levels may be modulated by altering the expression of TFs, either through overexpression or repression (Huang et al., 2010; Chen et al., 2015).

MYB proteins are TFs that play important roles in plant development and stress responses (Dubos et al., 2010). Sun et al. (2014) reported that a stress-responsive R2R3-type MYB gene of *P. trifoliata*, *PtsrMYB*, regulated its *ADC* gene, *PtADC*. Yeast one-hybrid assay demonstrated that *PtsrMYB* predominantly interacts with two regions of the *PtADC* promoter, indicating the *PtADC* may be a target gene of *PtsrMYB*. Moreover, overexpression of *PtsrMYB* led to an increase in mRNA levels of *ADC* genes in the transgenic lines when compared with WT plants, concurrent with increased PA levels. In a recent study, Chen et al. (2015) showed that overexpression of a cotton MYB TF, *GbMYB5*, also led to up-regulation of three polyamine biosynthetic genes in the transgenic lines. These findings suggest that MYBs might be likely to govern polyamine synthesis under abiotic stresses through regulating the relevant genes.

ABF is a key TF involved in the transduction of signals associated with drought and osmotic stress (Yoshida et al., 2010, 2015). It is known that under abiotic stress conditions, synthesis of ABA is typically increased, which in turn triggers signaling through a network that includes components such as ABA receptors, protein phosphatases, and SnRK proteins. The activated SnRK proteins can in turn phosphorylate ABF TFs, which then regulate downstream target genes (Danquah et al., 2014; Zhang et al., 2015a). In addition, PA biosynthetic genes, such as those encoding ADC, SAMDC, SPDS, have been shown to be induced under drought stress or following ABA treatment, and this is accompanied by increase in the endogenous PAs. However, whether the induction of PA genes or the accumulation of PAs is directly associated with ABA signaling cascades has not yet been addressed. Recently, PtrABF from *P. trifoliata*, an ABF4 homolog, was shown to regulate the expression of an *ADC* gene by interacting with the ABRE elements within the *ADC* promoter. Overexpression of *PtrABF* greatly increased the mRNA levels of *ADC*, and resulted in an increase in endogenous Put levels, whereas treatment of the *PtrABF*-overexpressing lines with an ADC inhibitor resulted in a decrease of Put contents, and compromised dehydration tolerance (Huang et al., 2010; Zhang et al., 2015a). These results provide convincing evidence that *ADC* is a candidate target gene of ABF. Characterization of this regulatory cascade may elucidate the transcriptional regulation of *ADC* genes and the associated accumulation of PAs under drought or osmotic stresses.

WRKY proteins comprise a large family of TFs in plants and play important roles in regulating the synthesis of several metabolites, such as lignin, phytoalexins, terpenoid indole alkaloid (Suttipanta et al., 2011). Recently, Gong et al. (2015) reported that overexpression of *FcWRKY70*, a WRKY70 homolog from *Fortunella crassifolia*, led to the increased expression of *ADC* genes, whereas suppression of *FcWRKY70*, using an RNAi approach, down-regulated *ADC* expression. Put levels were prominently increased in *FcWRKY70*-overexpressing lines, but decreased in the RNAi lines when compared to the WT. In addition, FcWRKY70 can interact with the W-box elements in the *ADC* promoter. Taken together, these results indicate that FcWRKY70 may also act as a positive regulator of *ADC* expression and regulate Put synthesis under abiotic stresses.

The above data suggests that *ADC* genes may be under the control of members from different TF families, including ABF, WRKY, and MYB proteins (Figure 1). However, TFs from other families may also be involved in the regulation of *ADC* gene expression (Huang et al., 2015). Of note, all of the TFs mentioned above are proposed as positive regulators of PA biosynthesis and *ADC* expression, while TFs that may negatively regulate PA gene expression have not been characterized. In addition, it is not known whether other PA biosynthetic genes, such as those encoding SAMDC, SPDS and SPMS proteins, are regulated by the same TFs mentioned above.

Apart from the TFs, other regulatory proteins may also control PA biosynthetic gene expression and PA accumulation. One of these is mitogen-activated protein kinase (MAPK), which has been shown to play roles in various signaling pathways related to plant development and stress responses. Huang et al. (2011) reported that the expression of *NtADC1* and *NtSAMDC*, two tobacco genes involved in PA biosynthesis, was induced in transgenic tobacco plants overexpressing *PtrMAPK* of trifoliate orange. It can be inferred from this result that MAPK may phosphorylate the two corresponding proteins, but this needs to be verified in the future.

## Perspectives and Concluding Remarks

Polyamines are considered to play important roles in protecting plant cells from stress-associated damages. To date, tremendous progresses have been made in understanding the significance of PAs in stress responses. There is accumulating evidence that PA levels undergo extensive changes in response to a range of abiotic stresses, and physiological, molecular and genetic approaches have been used to identify and functionally characterize PA biosynthetic genes in various plant species. These efforts underpin our understanding of the role of PAs in counteracting adverse environmental cues, and provide valuable information for enhancing stress tolerance through the modulation of cellular PA levels via exogenous PA application, or the transgenic manipulation of PA biosynthetic genes. Nevertheless, many key questions remain unanswered. First, the causal relationship between PA accumulation and stress tolerance has not been determined, despite numerous observations of changes in PA levels in response to abiotic stresses. Second, the cellular compartmentation and transportation of PAs is not well understood, although a few PA transporters have been identified (Fujita et al., 2012; Mulangi et al., 2012). In addition, the mode of action of PAs in enhancing stress tolerance has not been definitely established, although several possible models have been proposed. One example is the scarcity of direct evidence confirming the involvement of PAs in the activation of antioxidant enzymes for ROS detoxification. Last, the signaling cascades linking stress responses and PA genes are still far from being well defined. To date, TFs regulating *ADC* genes have been identified, but those that regulate other PA biosynthetic genes are unknown. In keeping with these unanswered questions, there are several promising areas of future study. First, the sites of PA production and actions in plant cells need to be identified and to this end, the cellular localization of PAs and their transporters should be determined. Second, the physiological and molecular mechanisms concerning the roles of PAs in stress tolerance need to be elucidated, and in particular, how PAs contribute to the activation of antioxidant enzymes and ROS removal should be clearly deciphered. Last but not the least, the molecular mechanisms underlying the accumulation of PAs in response to abiotic stresses, including the PA biosynthetic genes and the transcriptional regulation network associated with those genes, must be defined. This information will advance our understanding of PA accumulation and gene expression, and can be incorporated with physiological, biochemical, molecular and genetic approaches to better understand the complex regulation of PA synthesis under abiotic stresses, as well as the cross talk between different TF-mediated signaling pathways.

### Conflict of Interest Statement

The authors declare that the research was conducted in the absence of any commercial or financial relationships that could be construed as a potential conflict of interest.

## References

[B1] Ahou A. Martignago D. Alabdallah O. Tavazza R. Stano P. Macone A. (2014). A plant spermine oxidase/dehydrogenase regulated by the proteasome and polyamines. J. Exp. Bot. 65, 1585–1603. 10.1093/jxb/eru016 24550437

[B2] Alcázar R. Altabella T. Marco F. Bortolotti C. Reymond M. Koncz C. (2010a). Polyamines: molecules with regulatory functions in plant abiotic stress tolerance. Planta 231, 1237–1249. 10.1007/s00425-010-1130-0 20221631

[B3] Alcázar R. Planas J. Saxena T. Zarza X. Bortolotti C. Cuevas J. (2010b). Putrescine accumulation confers drought tolerance in transgenic *Arabidopsis* plants over-expressing the homologous Arginine decarboxylase 2 gene. Plant Physiol. Bioch. 48, 547–552. 10.1016/j.plaphy.2010.02.002 20206537

[B4] Angelini R. Cona A. Federico R. Fincato P. Tavladoraki P. Tisi A. (2010). Plant amine oxidases “on the move”: an update. Plant Physiol. Bioch. 48, 560–564. 10.1016/j.plaphy.2010.02.001 20219383

[B5] Bachrach U. (2010). The early history of polyamine research. Plant Physiol. Bioch. 48, 490–495. 10.1016/j.plaphy.2010.02.003 20219382

[B6] Basu S. Roychoudhury A. Sengupta D. N. (2014). Identification of transactingfactors regulating SamDC expression in *Oryza sativa*. Biochem. Biophys. Res. Commun. 445, 398–403. 10.1016/j.bbrc.2014.02.004 24530223

[B7] Biswas M. S. Mano J. (2015). Lipid peroxide-derived short-chain carbonyls mediate H_2_O_2_-induced and NaCl-induced programmed cell death in plants. Plant Physiol. 168, 885–898. 10.1104/pp.115.256834 26025050 PMC4741343

[B8] Capell T. Bassie L. Christou P. (2004). Modulation of the polyamine biosynthetic pathway in transgenic rice confers tolerance to drought stress. Proc. Natl. Acad. Sci. U.S.A. 101, 9909–9914. 10.1073/pnas.0306974101 15197268 PMC470772

[B9] Cervelli M. Cona A. Angelini R. Polticelli F. Federico R. Mariottini P. (2001). A barley polyamine oxidase isoform with distinct structural features and subcellular localization. Eur. J. Biochem. 268, 3816–3830. 10.1046/j.1432-1327.2001.02296.x 11432750

[B10] Cervelli M. Tavladoraki P. Agostino S. D. Angelini R. Federico R. Mariottini P. (2000). Isolation and characterization of three polyamine oxidase genes from *Zea mays*. Plant Physiol. Bioch. 38, 667–677. 10.1016/S0981-9428(00)01170-0

[B11] Chen T. Li W. Hu X. Guo J. Liu A. Zhang B. L. (2015). A cotton MYB transcription factor, GbMYB5, is positively involved in plant adaptive response to drought stress. Plant Cell Physiol. 56, 917–929. 10.1093/pcp/pcv019 25657343

[B12] Cona A. Rea G. Angelini R. Federico R. Tavladoraki P. (2006). Function of amine oxidases in plant development and defence. Trends Plant Sci. 11, 80–88. 10.1016/j.tplants.2005.12.009 16406305

[B13] Danquah A. de Zelicout A. Colcombet J. Hirt H. (2014). The role of ABA and MAPK signaling pathways in plant abiotic stress responses. Biotechnol. Adv. 32, 40–52. 10.1016/j.biotechadv.2013.09.006 24091291

[B14] Do P. T. Drechsel O. Heyer A. G. Hincha D. K. Zuther E. (2014). Changes in free polyamine levels, expression of polyamine biosynthesis genes, and performance of rice cultivars under salt stress: a comparison with responses to drought. Front. Plant Sci. 5:182. 10.3389/fpls.2014.00182 24847340 PMC4021140

[B15] Duan J. Li J. Guo S. Kang Y. (2008). Exogenous spermidine affects polyamine metabolism in salinity-stressed *Cucumis sativus* roots and enhances short-term salinity tolerance. J. Plant Physiol. 165, 1620–1635. 10.1016/j.jplph.2007.11.006 18242770

[B16] Dubos C. Stracke R. Grotewold E. Weisshaar B. Martin C. Lepiniec L. (2010). MYB transcription factors in *Arabidopsis*. Trends Plant Sci. 15, 573–581. 10.1016/j.tplants.2010.06.005 20674465

[B17] Espasandin F. D. Maiale S. J. Calzadilla P. Ruiz O. A. Sansberro P. A. (2014). Transcriptional regulation of 9-*cis*-epoxycarotenoid dioxygenase (NCED) gene by putrescine accumulation positively modulates ABA synthesis and drought tolerance in *Lotus tenuis* plants. Plant Physiol. Bioch. 76, 29–35. 10.1016/j.plaphy.2013.12.018 24448322

[B18] Fincato P. Moschou P. N. Spedaletti V. Tavazza R. Angelini R. Federico R. (2011). Functional diversity inside the *Arabidopsis* polyamine oxidase gene family. J. Exp. Bot. 62, 1155–1168. 10.1093/jxb/erq341 21081665

[B19] Fujita M. Fujita Y. Iuchi S. Yamada K. Kobayashi Y. Urano K. (2012). Natural variation in a polyamine transporter determines paraquat tolerance in *Arabidopsis*. Proc. Natl. Acad. Sci. U.S.A. 109, 6343–6347. 10.1073/pnas.1121406109 22492932 PMC3341036

[B20] Gehan M. A. Greenham K. Mockler T. C. McClung C. R. (2015). Transcriptional networks-crops, clocks, and abiotic stress. Curr. Opin. Plant Biol. 24, 39–46. 10.1016/j.pbi.2015.01.004 25646668

[B21] Gil-Amado J. A. Gomez-Jimenez M. C. (2012). Regulation of polyamine metabolism and biosynthetic gene expression during olive mature-fruit abscission. Planta 235, 1221–1237. 10.1007/s00425-011-1570-1 22167259

[B22] Gill S. S. Tuteja N. (2010). Polyamines and abiotic stress tolerance in plants. Plant Signal. Behav. 5, 26–33. 10.4161/psb.5.1.10291 20592804 PMC2835953

[B23] Gong X. Q. Zhang J. Y. Hu J. B. Wang W. Wu H. Zhang Q. H. (2015). FcWRKY70, a WRKY protein of *Fortunella crassifolia*, functions in drought tolerance and modulates putrescine synthesis by regulating arginine decarboxylase gene. Plant Cell Environ. 10.1111/pce.12539 25808564

[B24] Gupta K. Dey A. Gupta B. (2013). Plant polyamines in abiotic stress responses. Acta Physiol. Plant. 35, 2015–2036. 10.1007/s11738-013-1239-4

[B25] Hanfrey C. Sommer S. Mayer M. J. Burtin D. Michael A. J. (2001). *Arabidopsis* polyamine biosynthesis: absence of ornithine decarboxylase and the mechanism of arginine decarboxylase activity. Plant J. 27, 551–560. 10.1046/j.1365-313X.2001.01100.x 11576438

[B26] Harindra Champa W. A. Gilla M. I. S. Mahajanb B. V. C. Bedic S. (2015). Exogenous treatment of spermine to maintain quality and extend postharvest life of table grapes (*Vitis vinifera* L.) cv. Flame Seedless under low temperature storage. LWT Food Sci Technol. 60, 412–419. 10.1016/j.lwt.2014.08.044

[B27] Hatmi S. Gruau C. Trotel-Aziz P. Villaume S. Rabenoelina F. Baillieul F. (2015). Drought stress tolerance in grapevine involves activation of polyamine oxidation contributing to improved immune response and low susceptibility to *Botrytis cinerea*. J. Exp. Bot. 66, 775–787. 10.1093/jxb/eru436 25385768

[B28] Huang X. S. Huo T. Fu X. Z. Fan Q. J. Liu J. H. (2011). Cloning, molecular characterization of a mitogen-activated protein kinase gene from *Poncirus trifoliata* and its ectopic expression confers dehydration/drought tolerance in transgenic tobacco. J. Exp. Bot. 62, 5191–5206. 10.1093/jxb/err229 21778184 PMC3193021

[B29] Huang X. S. Liu J. H. Chen X. J. (2010). Overexpression of PtrABF gene, a bZIP transcription factor isolated from *Poncirus trifoliata*, enhances dehydration and drought tolerance in tobacco via scavenging ROS and modulating expression of stress-responsive genes. BMC Plant Biol. 10:230. 10.1186/1471-2229-10-230 20973995 PMC3017851

[B30] Huang X. S. Zhang Q. H. Zhu D. X. Fu X. Z. Wang M. Zhang Q. (2015). ICE1 of *Poncirus trifoliata* functions in cold tolerance by modulating polyamine levels through interacting with arginine decarboxylase. J. Exp. Bot. 66, 3259–3274. 10.1093/jxb/erv138 25873670 PMC4449543

[B31] Hussain S. S. Ali M. Ahmad M. Siddique K. H. (2011). Polyamines: natural and engineered abiotic and biotic stress tolerance in plants. Biotechnol. Adv. 29, 300–311. 10.1016/j.biotechadv.2011.01.003 21241790

[B32] Igarashi K. Kashiwagi K. (2010). Modulation of cellular function by polyamines. Intl. J. Biochem. Cell. Biol. 42, 39–51. 10.1016/j.biocel.2009.07.009 19643201

[B33] Ikbal F. E. Hernández J. A. Barba-Espín G. Koussa T. Aziz A. Faize M. (2014). Enhanced salt-induced antioxidative responses involve a contribution of polyamine biosynthesis in grapevine plants. J. Plant Physiol. 171, 779–788. 10.1016/j.jplph.2014.02.006 24877669

[B34] Kamada-Nobusada T. Hayashi M. Fukazawa M. Sakakibara H. Nishimura M. (2008). A putative peroxisomal polyamine oxidase, AtPAO4, is involved in polyamine catabolism in *Arabidopsis thaliana*. Plant Cell Physiol. 49, 1272–1282. 10.1093/pcp/pcn114 18703589

[B35] Kamiab F. Talaie A. Khezri S. Javanshah A. (2014). Exogenous application of free polyamines enhance salt tolerance of pistachio (*Pistacia vera* L.) seedlings. Plant Growth Regul. 72, 257–268. 10.1007/s10725-013-9857-9

[B36] Kasukabe Y. He L. Nada K. Misawa S. Ihara I. Tachibana S. (2004). Overexpression of spermidine synthase enhances tolerance to multiple environmental stresses and up-regulates the expression of various stress-regulated genes in transgenic *Arabidopsis thaliana*. Plant Cell Physiol. 45, 712–722. 10.1093/pcp/pch083 15215506

[B37] Kasukabe Y. He L. Watakabe Y. Otani M. Shimada T. Tachibana S. (2006). Improvement of environmental stress tolerance of sweet potato by introduction of genes for spermidine synthase. Plant Biotechnol. 23, 75–83. 10.5511/plantbiotechnology.23.75

[B38] Kim N. H. Kim B. S. Hwang B. K. (2013). Pepper arginine decarboxylase is required for polyamine and gamma-aminobutyric acid signaling in cell death and defense response. Plant Physiol. 162, 2067–2083. 10.1104/pp.113.217372 23784462 PMC3729783

[B39] Kitashiba H. Honda C. Moriguchi T. (2006). Identification of polyamine oxidase genes from apple and expression analysis during fruit development and cell growth. Plant Biotechnol. 23, 425–429. 10.5511/plantbiotechnology.23.425

[B40] Krishnamurthy R. Bhagwat K. A. (1989). Polyamines as modulators of salt tolerance in rice cultivars. Plant Physiol. 91, 500–504. 10.1104/pp.91.2.500 16667061 PMC1062029

[B41] Kumar R. R. Sharma S. K. Rai G. K. Singh K. Choudhury M. Dhawan G. (2014). Exogenous application of putrescine at pre-anthesis enhances the thermotolerance of wheat (*Triticum aestivum* L.). Indian J. Biochem. Biophys. 51, 396–406. 25630110

[B42] Kusano T. Berberich T. Tateda C. Takahashi Y. (2008). Polyamines: essential factors for growth and survival. Planta 228, 367–381. 10.1007/s00425-008-0772-7 18594857

[B43] Lee J. Sperandio V. Frantz D. E. Longgood J. Camilli A. Phillips M. A. (2009). An alternative polyamine biosynthetic pathway is widespread in bacteria and essential for biofilm formation in *Vibrio cholerae*. J. Biol. Chem. 284, 9899–9907. 10.1074/jbc.M900110200 19196710 PMC2665113

[B44] Liu H. P. Dong B. H. Zhang Y. Y. Liu Z. P. Liu Y. L. (2004). Relationship between osmotic stress and the levels of free, conjugated, and bound polyamines in leaves of wheat seedlings. Plant Sci. 166, 1261–1267. 10.1016/j.plantsci.2003.12.039

[B45] Liu J. H. Ban Y. Wen X. P. Nakajima I. Moriguchi T. (2009). Molecular cloning and expression analysis of an arginine decarboxylase gene from peach (*Prunus persica*). Gene 429, 10–17. 10.1016/j.gene.2008.10.003 18996450

[B46] Liu J. H. Inoue H. Moriguchi T. (2008). Salt stress-mediated changes in free polyamine titers and expression of genes responsible for polyamine biosynthesis of apple in vitro shoots. Environ. Exp. Bot. 62, 28–35. 10.1016/j.envexpbot.2007.07.002

[B47] Liu J. H. Kitashiba H. Wang J. Ban Y. Moriguchi T. (2007). Polyamines and their ability to provide environmental stress tolerance to plants. Plant Biotechnol. 24, 117–126. 10.5511/plantbiotechnology.24.117

[B48] Liu J. H. Nada K. Honda C. Kitashiba H. Wen X. P. Pang X. M. (2006). Polyamine biosynthesis of apple callus under salt stress: importance of the arginine decarboxylase pathway in stress response. J. Exp. Bot. 57, 2589–2599. 10.1093/jxb/erl018 16825316

[B49] Liu J. H. Nakajima I. Moriguchi T. (2011). Effects of salt and osmotic stresses on free polyamine content and expression of polyamine biosynthetic genes in *Vitis vinifera*. Biol. Plantarum 55, 340–344. 10.1007/s10535-011-0050-6

[B50] Liu J. H. Peng T. Dai W. S. (2014a). Critical *cis*-acting elements and interacting transcription factors: key players associated with abiotic stress responses in plants. Plant Mol. Biol. Rep. 32, 303–317. 10.1007/s11105-013-0667-z

[B51] Liu T. Kim D. W. Niitsu M. Maeda S. Watanabe M. Kamio Y. (2014b). Polyamine oxidase 7 is a terminal catabolism-type enzyme in *Oryza sativa* and is specifically expressed in anthers. Plant Cell Physiol. 55, 1110–1122. 10.1093/pcp/pcu047 24634478

[B52] Ma Y. Dai X. Xu Y. Luo W. Zheng X. Zeng D. (2015). COLD1 confers chilling tolerance in rice. Cell 160, 1209–1221. 10.1016/j.cell.2015.01.046 25728666

[B53] Marco F. Alcazar R. Tiburcio A. F. Carrasco P. (2011). Interactions between polyamines and abiotic stress pathway responses unraveled by transcriptome analysis of polyamine overproducers. OMICS 15, 775–781. 10.1089/omi.2011.0084 22011340 PMC3229227

[B54] Minocha R. Majumdar R. Minocha S. C. (2014). Polyamines and abiotic stress in plants: a complex relationship. Front. Plant Sci. 5:175. 10.3389/fpls.2014.00175 24847338 PMC4017135

[B55] Mitsuya Y. Takahashi Y. Berberich T. Miyazaki A. Matsumura H. Takahashi H. (2009). Spermine signaling plays a significant role in the defense response of *Arabidopsis thaliana* to cucumber mosaic virus. J. Plant Physiol. 166, 626–643. 10.1016/j.jplph.2008.08.006 18922600

[B56] Møller S. G. McPherson M. J. (1998). Developmental expression and biochemical analysis of the *Arabidopsis* atao1 gene encoding an H_2_O_2_-generating diamine oxidase. Plant J. 13, 781–791. 10.1046/j.1365-313X.1998.00080.x 9681017

[B57] Mo H. Wang X. Zhang Y. Zhang G. Zhang J. F. Ma Z. Y. (2015). Cotton polyamine oxidase is required for spermine and camalexin signalling in the defence response to *Verticillium dahliae*. Plant J. 83, 962–975. 10.1111/tpj.12941 26221980

[B58] Moschou P. N. Paschalidis K. A. Delis I. D. Andriopoulou A. H. Lagiotis G. D. Yakoumakis D. I. (2008). Spermidine exodus and oxidation in the apoplast induced by abiotic stress is responsible for H_2_O_2_ signatures that direct tolerance responses in tobacco. Plant Cell 20, 1708–1724. 10.1105/tpc.108.059733 18577660 PMC2483379

[B59] Moschou P. N. Wu J. Cona A. Tavladoraki P. Angelini R. Roubelakis-Angelakis K. A. (2012). The polyamines and their catabolic products are significant players in the turnover of nitrogenous molecules in plants. J. Exp. Bot. 63, 5003–5015. 10.1093/jxb/ers202 22936828

[B60] Mostofa M. G. Yoshida N. Fujita M. (2014). Spermidine pretreatment enhances heat tolerance in rice seedlings through modulating antioxidative and glyoxalase systems. Plant Growth Regul. 73, 31–44. 10.1007/s10725-013-9865-9

[B61] Mulangi V. Phuntumart V. Aouida M. Ramotar D. Morris P. (2012). Functional analysis of OsPUT1, a rice polyamine uptake transporter. Planta 235, 1–11. 10.1007/s00425-011-1486-9 21796369

[B62] Nakashima K. Ito Y. Yamaguchi-Shinozaki K. (2009). Transcriptional regulatory networks in response to abiotic stresses in *Arabidopsis* and grasses. Plant Physiol. 149, 88–95. 10.1104/pp.108.129791 19126699 PMC2613698

[B63] Ono Y. Kim D. W. Watanabe K. Sasaki A. Niitsu M. Berberich T. (2012). Constitutively and highly expressed *Oryza sativa* polyamine oxidases localize in peroxisomes and catalyze polyamine back conversion. Amino Acids 42, 867–876. 10.1007/s00726-011-1002-3 21796433

[B64] Pegg A. E. Casero R. A. Jr. (2011). Current status of the polyamine research field. Methods Mol. Biol. 720, 3–35. 10.1007/978-1-61779-034-8_1 21318864 PMC3652263

[B65] Pillai M. A. Akiyama T. (2004). Differential expression of an *S*-adenosyl-L-methionine decarboxylase gene involved in polyamine biosynthesis under low temperature stress in japonica and indica rice genotypes. Mol. Genet. Genomics 271, 141–149. 10.1007/s00438-003-0963-7 14727183

[B66] Planas-Portell J. Gallart M. Tiburcio A. F. Altabella T. (2013). Copper-containing amine oxidases contribute to terminal polyamine oxidation in peroxisomes and apoplast of *Arabidopsis thaliana*. BMC Plant Biol. 13:109. 10.1186/1471-2229-13-109 23915037 PMC3751259

[B67] Radhakrishnan R. Lee I. J. (2013). Spermine promotes acclimation to osmotic stress by modifying antioxidant, abscisic acid, and jasmonic acid signals in soybean. J. Plant Growth Regul. 32, 22–30. 10.1007/s00344-012-9274-8

[B68] Rea G. Laurenzi M. Tranquilli E. D’Ovidio R. Federico R. Angelini R. (1998). Developmentally and wound-regulated expression of the gene encoding a cell wall copper amine oxidase in chickpea seedlings. FEBS Lett. 437, 177–182. 10.1016/S0014-5793(98)01219-8 9824285

[B69] Richards F. J. Coleman R. G. (1952). Occurrence of putrescine in potassium deficient barley. Nature 170, 479–481. 10.1038/170460a0 12993199

[B70] Roy M. Wu R. (2001). Arginine decarboxylase transgene expression and analysis of environmental stress tolerance in transgenic rice. Plant Sci. 160, 869–875. 10.1016/S0168-9452(01)00337-5 11297783

[B71] Roy M. Wu R. (2002). Overexpression of *S*-adenosylmethionine decarboxylase gene in rice increases polyamine level and enhances sodium chloride-stress tolerance. Plant Sci. 163, 987–992. 10.1016/S0168-9452(02)00272-8

[B72] Santa-Cruz A. Perez-Alfocea F. Caro M. Acosta M. (1998). Polyamines as short-term salt tolerance traits in tomato. Plant Sci. 138, 9–16. 10.1016/S0168-9452(98)00143-5

[B73] Seki M. Narusaka M. Ishida J. Nanjo T. Fujita M. Oono Y. (2002). Monitoring the expression profiles of ca. 7000 *Arabidopsis* genes under drought, cold, and high-salinity stresses using a full-length cDNA microarray. Plant J. 31, 279–292. 10.1046/j.1365-313X.2002.01359.x 12164808

[B74] Shi H. Chan Z. (2014). Improvement of plant abiotic stress tolerance through modulation of the polyamine pathway. J. Intg. Plant Biol. 56, 114–121. 10.1111/jipb.12128 24401132

[B75] Shi J. Fu X. Z. Peng T. Huang X. S. Fan Q. J. Liu J. H. (2010). Spermine pretreatment confers dehydration tolerance of citrus in vitro plants via modulation of antioxidative capacity and stomatal response. Tree Physiol. 30, 914–922. 10.1093/treephys/tpq030 20462936

[B76] Shinozaki K. Yamaguchi-Shinozaki K. (2007). Gene networks involved in drought stress response and tolerance. J. Exp. Bot. 58, 221–227. 10.1093/jxb/erl164 17075077

[B77] Shi Y. Ding Y. Yang S. (2015). Cold signal transduction and its interplay with phytohormones during cold acclimation. Plant Cell Physiol. 56, 7–15. 10.1093/pcp/pcu115 25189343

[B78] Shu S. Yuan L. Y. Guo S. R. Sun J. Yuan Y. H. (2013). Effects of exogenous spermine on chlorophyll fluorescence, antioxidant system and ultrastructure of chloroplasts in *Cucumis sativus* L. under salt stress. Plant Physiol. Bioch. 63, 209–216. 10.1016/j.plaphy.2012.11.028 23291654

[B79] Silveira V. De Vita A. M. Macedo A. F. Dias M. F. R. Floh E. I. S. Santa- Catarina C. (2013). Morphological and polyamine content changes in embryogenic and non-embryogenic callus of sugarcane. Plant Cell Tiss. Organ Cult. 114, 351–364. 10.1007/s11240-013-0330-2

[B80] Suttipanta N. Pattanaik S. Kulshrestha M. Patra B. Singh S. K. Yuan L. (2011). The transcription factor CrWRKY1 positively regulates the terpenoid indole alkaloid biosynthesis in *Catharanthus roseus*. Plant Physiol. 157, 2081–2093. 10.1104/pp.111.181834 21988879 PMC3327198

[B81] Sun P. P. Zhu X. F. Huang X. S. Liu J. H. (2014). Overexpression of a stress-responsive MYB transcription factor of *Poncirus trifoliata* confers enhanced dehydration tolerance and increases polyamine biosynthesis. Plant Physiol. Bioch. 78, 71–79. 10.1016/j.plaphy.2014.02.022 24636909

[B82] Tanou G. Ziogas V. Belghazi M. Christou A. Filippou P. Job D. (2014). Polyamines reprogram oxidative and nitrosative status and the proteome of citrus plants exposed to salinity stress. Plant Cell Environ. 37, 864–885. 10.1111/pce.12204 24112028

[B83] Tavladoraki P. Rossi M. N. Saccuti G. Perez-Amador M. A. Polticelli F. Angelini R. (2006). Heterologous expression and biochemical characterization of a polyamine oxidase from *Arabidopsis* involved in polyamine back conversion. Plant Physiol. 141, 1519–1532. 10.1104/pp.106.080911 16778015 PMC1533960

[B84] Thomashow M. F. (2010). Molecular basis of plant cold acclimation: insights gained from studying the CBF cold response pathway. Plant Physiol. 154, 571–577. 10.1104/pp.110.161794 20921187 PMC2948992

[B85] Tiburcio A. F. Altabella T. Bitrián M. Alcázar R. (2014). The roles of polyamines during the lifespan of plants: from development to stress. Planta 240, 1–18. 10.1007/s00425-014-2055-9 24659098

[B86] Tisi A. Federico R. Moreno S. Lucretti S. Moschou P. N. Roubelakis-Angelakis K. A. (2011). Perturbation of polyamine catabolism can strongly affect root development and xylem differentiation. Plant Physiol. 157, 200–215. 10.1104/pp.111.173153 21746808 PMC3165870

[B87] Tuskan G. A. DiFazio S. Jansson S. Bohlmann J. Grigoriev I. Hellsten U. (2006). The genome of black cottonwood, *Populus trichocarpa* (Torr. & Gray). Science 313, 1596–1604. 10.1126/science.1128691 16973872

[B88] Urano K. Yoshiba Y. Nanjo T. Ito T. Yamaguchi-Shinozaki K. Shinozaki K. (2004). *Arabidopsis* stress-inducible gene for arginine decarboxylase AtADC2 is required for accumulation of putrescine in salt tolerance. Biochem. Biophys. Res. Commun. 313, 369–375. 10.1016/j.bbrc.2003.11.119 14684170

[B89] Vera-Sirera F. Minguet E. G. Singh S. K. Ljung K. Tuominen H. Blazquez M. A. (2010). Role of polyamines in plant vascular development. Plant Physiol. Bioch. 48, 534–539. 10.1016/j.plaphy.2010.01.011 20137964

[B90] Waie B. Rajam M. V. (2003). Effect of increased polyamine biosynthesis on stress responses in transgenic tobacco by introduction of human *S*-adenosylmethionine gene. Plant Sci. 164, 727–734. 10.1016/S0168-9452(03)00030-X

[B91] Wang B. Q. Zhang Q. F. Liu J. H. Li G. H. (2011a). Overexpression of PtADC confers enhanced dehydration and drought tolerance in transgenic tobacco and tomato: effect on ROS elimination. Biochem. Biophys. Res. Commun. 413, 10–16. 10.1016/j.bbrc.2011.08.015 21871871

[B92] Wang J. Sun P. P. Chen C. L. Wang Y. Fu X. Z. Liu J. H. (2011b). An arginine decarboxylase gene PtADC from *Poncirus trifoliata* confers abiotic stress tolerance and promotes primary root growth in *Arabidopsis*. J. Exp. Bot. 62, 2899–2914. 10.1093/jxb/erq463 21282323

[B93] Wang J. Liu J. H. Yu A. Xiang Y. Kurosawa T. Nada K. (2010). Cloning, biochemical identification, and expression analysis of a gene encoding *S*-adenosylmethionine decarboxylase in *Citrus sinensis* Osbeck. J. Hortic. Sci. Biotechnol. 85, 219–226.

[B94] Wang J. Liu J. H. (2009). Change in free polyamine contents and expression profiles of two polyamine biosynthetic genes in citrus embryogenic callus under abiotic stresses. Biotechnol. Biotechnol. Eq. 29, 1289–1293. 10.1080/13102818.2009.10817655

[B95] Wang W. Liu J. H. (2015). Genome-wide identification and expression analysis of the polyamine oxidase gene family in sweet orange (*Citrus sinensis*). Gene 555, 421–429. 10.1016/j.gene.2014.11.042 25445392

[B96] Wang W. Vinocur B. Altman A. (2003). Plant responses to drought, salinity and extreme temperatures: towards genetic engineering for stress tolerance. Planta 218, 1–14. 10.1007/s00425-003-1105-5 14513379

[B97] Wen X. P. Ban Y. Inoue H. Matsuda N. Moriguchi T. (2009). Spermidine levels are implicated in heavy metal tolerance in a spermidine synthase overexpressing transgenic European pear by exerting antioxidant activities. Transgenic Res. 19, 91–103. 10.1007/s11248-009-9296-6 19544002

[B98] Wi S. J. Kim W. T. Park K. Y. (2006). Overexpression of carnation *S*-adenosylmethionine decarboxylase gene generates a broad spectrum tolerance to abiotic stresses in transgenic tobacco plants. Plant Cell Rep. 25, 1111–1121. 10.1007/s00299-006-0160-3 16642382

[B99] Wisniewski M. Nassuth N. Teulières C. Marque C. Rowland J. Cao P. B. (2014). Genomics of cold hardiness in woody plants. Crit. Rev. Plant Sci. 33, 92–124. 10.1080/07352689.2014.870408

[B100] Xu S. Hu J. Li Y. Ma W. Zheng Y. Zhu S. J. (2011). Chilling tolerance in *Nicotiana tabacum* induced by seed priming with putrescine. Plant Growth Regul. 63, 279–290. 10.1007/s10725-010-9528-z

[B101] Yang J. C. Zhang J. H. Liu K. Wang Z. Q. Liu L. J. (2007). Involvement of polyamines in the drought resistance of rice. J. Exp. Bot. 58, 1545–1555. 10.1093/jxb/erm032 17332417

[B102] Yin L. Wang S. Tanaka K. Fujihara S. Itai A. Den X. (2015). Silicon-mediated changes in polyamines participate in silicon-induced salt tolerance in *Sorghum bicolor* L. Plant Cell Environ. 10.1111/pce.12521 [Epub ahead of print]. 25753986

[B103] Yoda H. Hiroi Y. Sano H. (2006). Polyamine oxidase is one of the key elements for oxidative burst to induce programmed cell death in tobacco cultured cells. Plant Physiol. 142, 193–206. 10.1104/pp.106.080515 16844838 PMC1557616

[B104] Yoshida T. Fujita Y. Maruyama K. Mogami J. Todaka D. Shinozaki K. (2015). Four *Arabidopsis* AREB/ABF transcription factors function predominantly in gene expression downstream of SnRK2 kinases in abscisic acid signalling in response to osmotic stress. Plant Cell Environ. 38, 35–49. 10.1111/pce.12351 24738645 PMC4302978

[B105] Yoshida T. Fujita Y. Sayama H. Kidokoro S. Maruyama K. Mizoi J (2010). AREB1, AREB2, and ABF3 are master transcription factors that cooperatively regulate ABRE-dependent ABA signaling involved in drought stress tolerance and require ABA for full activation. Plant J. 61, 672–685. 10.1111/j.1365-313X.2009.04092.x 19947981

[B106] Yuan L. Liu X. Luo M. Yang S. Wu K. (2013). Involvement of histone modifications in plant abiotic stress responses. J. Intg. Plant Biol. 55, 892–901. 10.1111/jipb.12060 24034164

[B107] Zhang Q. H. Wang M. Hu J. B. Wang W. Fu X. Z. Liu J. H. (2015a). PtrABF of *Poncirus trifoliata* functions in dehydration tolerance by reducing stomatal density and maintaining reactive oxygen species homeostasis. J. Exp. Bot. 66, 5911–5927. 10.1093/jxb/erv301 26116025 PMC4566982

[B108] Zhang Y. Zhang H. Zou Z. R. Liu Y. Hu X. H. (2015b). Deciphering the protective role of spermidine against saline-alkaline stress at physiological and proteomic levels in tomato. Phytochemistry 110, 13–21. 10.1016/j.phytochem.2014.12.021 25579998

